# Knockout RAGE alleviates cardiac fibrosis through repressing endothelial-to-mesenchymal transition (EndMT) mediated by autophagy

**DOI:** 10.1038/s41419-021-03750-4

**Published:** 2021-05-11

**Authors:** Lu Zhang, Jiaqi He, Junyan Wang, Jing Liu, Zixin Chen, Bo Deng, Lan Wei, Hanqin Wu, Birong Liang, Huan Li, Yusheng Huang, Lu Lu, Zhongqi Yang, Shaoxiang Xian, Lingjun Wang

**Affiliations:** 1 The First Affiliated Hospital, Guangzhou University of Chinese Medicine, Guangzhou, 510405 China; 2 The First Clinical Medical School, Guangzhou University of Chinese Medicine, Guangzhou, 510405 China; 3 Lingnan Medical Research Center, Guangzhou University of Chinese Medicine, Guangzhou, 510405 China; 4 Guangzhou Key Laboratory of Chinese Medicine for Prevention and Treatment of Chronic Heart Failure, Guangzhou, 510405 China; 5 National Clinical Research Base of Traditional Chinese Medicine, Guangzhou, 510405 China

**Keywords:** Heart failure, Experimental models of disease

## Abstract

Endothelial-to-mesenchymal transition (EndMT) has been shown to contribute to cardiac fibrosis and heart failure (HF). Recent studies have demonstrated that EndMT is regulated by autophagy, and we previously showed suppression of excessive autophagy and alleviation of cardiac fibrosis in HF mice with inactivated receptor for advanced glycation end products (RAGE). Thus, we investigated whether reduced cardiac fibrosis due to RAGE knockout occurred by inhibiting EndMT mediated by excessive autophagy. We found a decrease in endothelial cells (CD31^+^/VE-Cadherin^+^) and an increase in cells co-expressing CD31 and α-smooth muscle actin (α-SMA, myofibroblast marker) at 8 weeks in heart tissue of mice subjected to transverse aortic constriction (TAC), which implied EndMT. Knockout RAGE decreased EndMT accompanied by decreased expression of autophagy-related proteins (LC3BII/I and Beclin 1), and alleviated cardiac fibrosis and improved cardiac function in TAC mice. Moreover, 3-methyladenine (3-MA) and chloroquine (CQ), inhibitors of autophagy, attenuated EndMT, and cardiac fibrosis in TAC mice. Importantly, EndMT induced by AGEs could be blocked by autophagy inhibitor in vivo and in vitro. These results suggested that AGEs/RAGE-autophagy-EndMT axis involved in the development of cardiac fibrosis and knockout RAGE ameliorated cardiac fibrosis through decreasing EndMT regulated by autophagy, which could be a promising therapeutic strategy for HF.

## Introduction

Heart failure (HF) occurs as the final stage of many cardiovascular diseases and is a major health challenge worldwide. Cardiac remodeling is the process of structural and functional changes in the left ventricle and is associated with hypertrophy and apoptosis of cardiomyocytes, particularly ventricular fibrosis^1^, which is pivotal in developing HF^2,3^. The ventricular fibrotic process is initially triggered by the differentiation of cardiac fibroblasts into myofibroblasts. These activated fibroblasts display increased proliferative capacity and secrete large amounts of extracellular matrix (ECM)^4,5^. Resident proliferation of fibroblasts was traditionally considered as the origin of myofibroblasts^2–5^, but substantial evidence indicates myofibroblasts may also originate from other cellular sources. In many fibrotic conditions, myofibroblasts may stem from endothelial cells through endothelial-to-mesenchymal transition (EndMT)^6–9^. EndMT involves an intricate cellular differentiation process in which endothelial cells detach and migrate away from the endothelium, lose endothelial properties, and acquire mesenchymal features^10^. An increasing number of studies indicate EndMT is a common and potentially disease-causing process^11–15^ and contributes to cardiac fibrosis^16^. mRNA levels of EndMT-related genes are increased in the human fibrotic heart tissue^17^, and repression of EndMT attenuates isoproterenol-induced cardiac fibrosis^18^. Therefore, understanding the mechanism of EndMT is essential to potentially prevent cardiac fibrosis and HF.

EndMT is regulated by autophagy^19–21^, which plays an important role in many physiological and pathological processes^22,23^. Autophagy can be divided into three types in general. Macroautophagy is the most important, and is initiated by the formation of the phagophore, followed by the completion of a double-membrane autophagosome, then, autolysosome is formed through autophagosome dock and fuse with the lysosome, finally, acid hydrolases inside the autolysosome break down and degrade the autophagosome inner membrane and cargo^24^. Autophagy has been considered as a protective mechanism^25^ and is critical for the survival of cardiomyocytes subjected to stress and hypoxia^26–28^. Nevertheless, excessive autophagy is deleterious^19^ and accentuates cardiac fibrosis^29^. The increased autophagy is detrimental during reperfusion that causes significant cardiac injury^27^. We previously showed that excessive autophagy could be inhibited by deletion of the receptor for advanced glycation end products (RAGE), resulting in amelioration of cardiac fibrosis in HF^30^.

RAGE is the receptor of advanced glycation end products (AGEs). Increased AGEs and RAGE activate autophagy-associated signal pathways and induce autophagy in various diseases^30–33^. Recently, AGEs and their soluble receptor (sRAGE) have been confirmed as poor prognostic biomarkers of HF in non-diabetic patients^34^. AGEs/RAGE can also transduce fibrotic pathways^30,35^. Additionally, AGEs are triggers in EndMT, and AGEs/RAGE mediated EndMT in human endothelial cells^36^. Therefore, we aimed to explore whether the reduction of cardiac fibrosis by knockout RAGE occurred through inhibition of autophagy-regulated EndMT in HF mice induced by transverse aortic constriction (TAC).

## Materials and methods

### Animals

All experimental animal protocols for this study were approved by the Animal Care Committee of Guangzhou University of Chinese Medicine and were strictly in accordance with the National Institutes of Health Guidelines for Care and Use of Laboratory Animals.

Eight-week-old C57BL/6J wild-type male mice weighing 18–22 g were obtained from the Laboratory Animal Center of Guangzhou University of Chinese Medicine. RAGE knockout mice were a gift from Kanazawa University, Japan^37^. Mice were randomly assigned into eight groups: Sham, Sham + AGEs, Sham + RAGE^−/−^, TAC, TAC + AGEs, TAC + RAGE^−/−^, TAC + 3-methyladenine (3-MA), and TAC + chloroquine (CQ). From 4 weeks postoperatively, Sham + AGEs and TAC + AGEs mice were generated by intraperitoneal (i.p.) injection of AGEs (10 mg/kg/day); TAC + 3-MA and TAC + CQ mice were generated by i.p. injection of 3-MA (15 mg/kg/day, dissolved in 5% DMSO and 95% saline, Cat. M9281, Sigma, Saint Louis, MO, USA) and CQ (20 mg/kg/day, dissolved in 5% DMSO and 95% saline, Cat.C6628, Sigma). Sham and TAC groups were administered 0.9% sodium chloride. All animals were sacrificed for analysis at 8 weeks.

### Mouse model of TAC

Mice were anaesthetized by an i.p. injection of pentobarbital sodium (50 mg/kg, Sigma). Sham-operated mice underwent a thoracotomy procedure without the constriction of the aorta. In the TAC group, we used a 6–0 silk suture ligature to constrict transverse aorta against a blunted 27-gauge needle to yield a narrowing to 25–30% of its original cross-sectional area^38^.

### Echocardiography

Echocardiography was performed 8 weeks postoperatively by using a Vevo 2100 Imaging System (VisualSonics Inc., Toronto, ON, Canada) in mice under anesthesia with 1–2.5% isoflurane (RWD Life Science Co., Guangdong, China) inhalation. The M-mode echocardiogram of the mid-ventricle was examined in short-axis view. Digital images were analyzed in a blinded manner. Cardiac function indices included left ventricular ejection fraction (LVEF%), left ventricular fractional shortening (LVFS%), end-diastolic left ventricular internal dimension (LVIDd), end-systolic left ventricular internal dimension (LVIDs), left ventricular end-diastolic volume (LVEDV), and left ventricular end-systolic volume (LVESV).

### Histological examination of cardiac fibrosis

Heart tissue was harvested and rinsed with cold phosphate-buffered saline (PBS), fixed in 4% paraformaldehyde over 24 h at room temperature, then dehydrated and embedded in paraffin. Five-µm-thick slices were cut at the papillary muscle level for staining. Masson’s trichrome staining was scanned by using Caseviewer 2.0 (Panoramic 250/MIDI, 3D HISTECH, Budapest, Hungary). The Sirius red staining was photographed under polarized light. Quantification of all data was done using Image J (National Institutes of Health, Bethesda, MD, USA) software. At least nine fields per slides of six tissues sections per mouse were analyzed.

### Transmission electron microscopy (TEM)

Left ventricular tissue was harvested and cut into 1 mm × 1 mm × 1 mm pieces. Samples were fixed in 2.5% glutaraldehyde over 2 h at room temperature and transferred to 4 °C. After transferred to 1% osmium PBS buffer for 2 h, the samples were successively placed in ascending series of ethanol (30, 50, 70, 80, 95, 100, and 100%), 15 min each. Then permeated overnight with a 1:1 mixture of acetone, followed by soaking in 812 embedding agents at 60 °C for 48 h. The slices were 60-nm thick. Uranium-lead double staining was performed, and sections were imaged by TEM (JEOL 1400 Plus TEM system, JEOL Inc., Peabody, MA, USA) at 120 kV. The total autolysosomes were quantified according to the average value of nine fields beyond with six tissue sections in each group.

### Flow cytometry

Heart tissue was minced and placed into 60% collagenase II (A004174, Sangon Biotech, Shanghai, China) in Hanks’ solution with Ca^2+^ and Mg^2+^ (H1025, Solarbio, Beijing, China), and shaken at 37 °C for 20 min. Subsequently, the cells were passed through 70-µm nylon mesh and centrifuged (15 min, 300 × *g*, 4 °C). Cells were stained with APC-labeled anti-VE-cadherin (vascular endothelial cadherin) (EB-50-1441-82), PE/Cy7-labeled anti-CD31 (EB-25-0311-82), and FITC-labeled anti-α-SMA (EB-53-9760-82) for 20 min at room temperature. Finally, the stained single cells were resuspended in staining buffer and analyzed using a flow cytometer (MoFloAstrios EQs, BECKMAN, Bria, California, USA).

### Cell culture and adenovirus shRNA preparation and application

HUVECs (Cat.8000) were purchased from ScienCell Research Laboratories (Carlsbad, CA, USA) and cultured in ECM medium (Cat.1001) supplemented with 5% fetal bovine serum (Cat.0025), 1% endothelial cell growth supplement (Cat.1052), and 1% penicillin/streptomycin solution (Cat.0503) in a humidified 5% CO_2_ incubator at 37 °C. On day 4, HUVECs were treated with AGEs (Cat.2221-10, BioVision, Milpitas, CA, USA) at 50, 100, 200, or 400 µg/ml for 24 h and AGEs at 200 µg/ml for 6, 12, 24, and 48 h.

Adenoviruses with humanBECN1 (Ad-SiBECN1) were constructed by Genechem (Shanghai, China). Twenty-four hours after transfection, the adenovirus was removed, and transfection efficiency of Ad-SiBECN1 was evaluated by GFP fluorescent protein in the cells.

### Immunofluorescence staining

In vivo, after paraffin sections were dewaxed and dehydrated using xylene and graded ethanol series, antigens were retrieved by sodium citrate heating. Endogenous peroxidase was removed by adding 30% H_2_O_2_. An immunohistochemistry pen was used to draw a circle around the tissue. The sections were incubated with 0.3% Triton X-100/PBS at room temperature for 20 min and incubated with mouse IgG-blocking solution (M.O.M Kit, Vector Laboratories, Burlingame, CA, USA) diluted in 0.01% Triton X-100/PBS at room temperature for 1 h, after 5% goat serum (SL038, Solarbio, Beijing, China) was added to block the tissue for 30 min at room temperature. Subsequently, the sections were incubated overnight at 4 °C with the primary anti-CD31 antibody (ab28364, 1:60, Abcam, Cambridge, UK) and anti-α-SMA antibody (Abcam, ab7817, 1:200). After washing three times with PBS each for 3 min, secondary fluorescent antibodies were prepared at a ratio of 1:1000. The sections were incubated in the dark for 1 h at room temperature and then washed with PBS three times for 3 min each. Finally, DAPI solution was added to the sections for 5 min followed by three PBS washes.

In vitro, the harvested cells were fixed in 4% paraformaldehyde at room temperature, then incubated with 0.1% Triton X-100/PBS at room temperature for 20 min, and incubated with 5% goat serum (SL038, Solarbio) for 1 h at room temperature. The cells were incubated with primary anti-CD31 antibody (Abcam, ab28364, 1:200) and anti-α-SMA antibody (Abcam, ab7817, 1:200) at 4 °C overnight. The incubation method with secondary antibody and DAPI was same as described above for the tissue samples.

A confocal scanning microscope was used to image the slices in a dark room.

### Quantitative real-time PCR (qPCR)

Total RNA was extracted from the left ventricular tissue and cells by RNAzol RT (Molecular Research Center, Cincinnati, OH, USA). First-strand cDNA was synthesized with a FastKing RT Kit (Tiangen, Beijing, China). The cDNA was used to perform quantitative PCR on a CFX96 Real-Time System (Bio-Rad Laboratories, Inc., Hercules, CA, USA) using the SYBR Select master mix kit (Applied Biosystems, Austin, TX, USA). The PCR conditions were 2 min at 50 °C and 30 s at 95 °C followed by 40 cycles of 95 °C for 15 s, 60 °C for 1 min, and 15 s at 65 °C. The specific primers used are listed in Table 1. Relative amounts of mRNA for specific genes were calculated using 2^−ΔΔCt^ values. Each sample was run in duplicate, and the mean value of each set of duplicates normalized to that of mouse or human GAPDH was used to calculate relative gene expression.

**Table 1:** Primer sequence.

Gene	Forward sequence	Reverse sequence
m-GAPDH	GGCTGTATTCCCCTCCATCG	CCAGTTGGTAACAATGCCATGT
m-COL I	TGGCCTTGGAGGAAACTTTG	CTTGGAAACCTTGTGGACCAG
m-COL III	CTGTAACATGGAAACTGGGGAAA	CCATAGCTGAACTGAAAACCACC
m-CD31	ACCGGGTGCTGTTCTATAAGG	TCACCTCGTACTCAATCGTGG
m-VE-Cadherin	CACTGCTTTGGGAGCCTTC	GGGGCAGCGATTCATTTTTCT
m-α-SMA	GTCCCAGACATCAGGGAGTAA	TCGGATACTTCAGCGTCAGGA
m-N-Cadherin	AGCGCAGTCTTACCGAAGG	TCGCTGCTTTCATACTGAACTTT
H-GAPDH	CCATGGAGAAGGCTGGGG	CAAAGTTGTCATGGATGACC
H- CD31	GCGAGTCATGGCCCGAAGGC	GGTGGTGCTGACATCCGCGA
H-α-SMA	CTATGAGGGCTATGCCTTGCC	GCTCAGCCAGTAGTAACGAAGGA
H-VE-Cadherin	GTTCACCTTCTGCGAGGATATG	GATGGTGAGGATGCAGAGTAAG
H-N-Cadherin	TCAGGCGTCTGTAGAGGCTT	ATGCACATCCTTCGATAAGACTG

### Western blot

Left ventricular tissue was harvested and lysed using a Minute^TM^ Total Protein Extraction Kit for Cultured Cells and Tissues (Cat.SD-001/SN-002, Invent Biotechnologies, Plymouth, MN, USA). After denature proteins fully, lysates were subjected to SDS-PAGE and transferred to 0.45 µm polyvinylidene fluoride membranes (Cat.1620260, Bio-Rad Laboratories, Inc.). Membranes were blocked with 5% skim milk (Cat.9999, Cell Signaling Technology [CST], Danvers, MN, USA) at room temperature for 1 h and incubated overnight at 4 °C with 1:1000 GAPDH (CST, 2118L), 1:1000 RAGE (Abcam, 3611), 1:1000 Beclin 1 (CST, 3495S), 1:1000 LC3B (CST, 2775S), 1:500 CD31 (Abcam, 28364), 1:1000 α-SMA (Abcam, 7817), 1:1000 VE-Cadherin (Abcam, 33168), 1:1000 N-Cadherin (Proteintech, Rosemont, IL, USA, 22018-1-AP), and 1:1000 Collagen type I (COL 1) (Proteintech,14695-1-AP). Membranes were then incubated with HRP-conjugated secondary antibody (CST, 7074 or 7076) for 1 h at room temperature. Finally, the densities of the protein bands were quantified using Immobilon Western Chemiluminescent HRP Substrate (WBKLS0500, Millipore, Bedford, MA, USA) and visualized using a chemiluminescence system (Bio-Rad Laboratories, Inc.).

### Collagen gel contraction assay

The cell contraction assay was performed using the cell contraction assay kit (CBA-201, Cell Biolabs, San Diego, CA, US). Briefly, HUVECs transfected with or without Ad-SiBECN1 and Ad-SiRAGE were harvested and resuspended in medium. Collagen Gel Mix was prepared according to manufacturer protocol on ice. Next, HUVECs were resuspended at a concentration of 2 × 10^6^ cells/ml. An amount of 200 µl cell suspension was mixed with 800 µl collagen gel, after vigorous vortex, the total 1 ml cell suspension was added into each well of a 12-well tissue culture plate and incubated at 37 °C for 1 h. The control group, Ad-SiRAGE, and Ad-siBECN1 group were added to 1 ml culture medium, and others were added to 1 ml culture medium containing 200 µg/ml AGEs. Photographs were taken at 0 and 24 h to quantitate the gel contraction area ratio using Image J software.

### Statistical analysis

Values are presented as mean ± standard error of mean. Differences between experimental groups were determined by one-way or two-way ANOVA, followed by post hoc Fisher Tukey’s multiple comparison test. Analyses were carried out with Prism 7 (GraphPad, San Diego, CA, USA). *p* < 0.05 was considered statistically significant.

## Results

### RAGE knockout improves cardiac function at 8 weeks after TAC

**Fig. 1:**
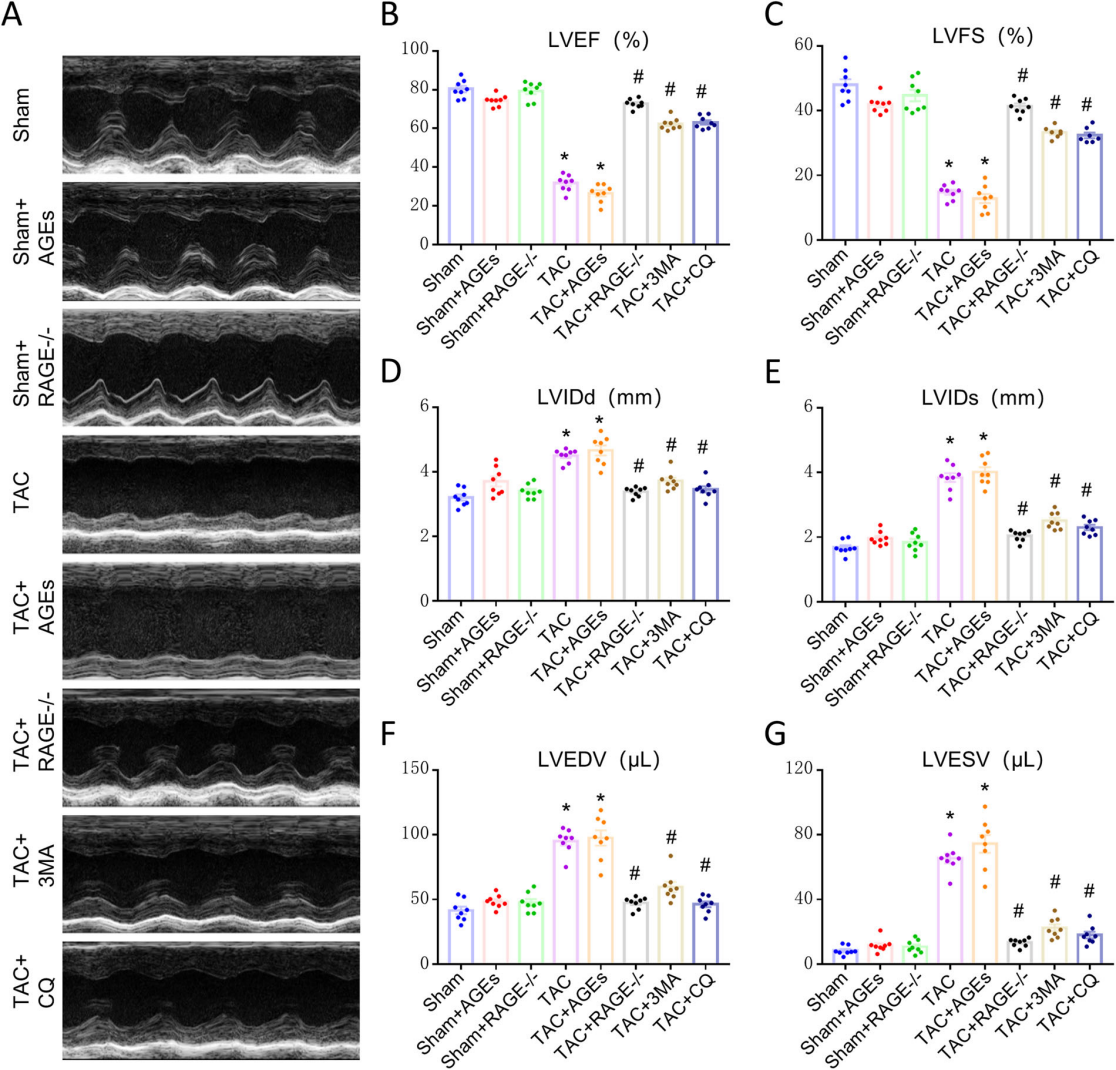
Improvement of cardiac function at 8 weeks after TAC by genetic deletion of RAGE or an autophagy inhibitor (3-MA or CQ). **A** Representative M-mode images. Echocardiography was performed to measure LVEF (**B**), LVFS (**C**), LVIDd (**D**), LVIDs (**E**), LVEDV (**F**), and LVESV (**G**). *n* = 8. Data are presented as mean ± SEM. *p < 0.05 vs. sham group, ^#^*p* < 0.05 vs. TAC group.

To clarify the potential influence of AGEs/RAGE on HF, we examined cardiac function at 8 weeks. The LVEF and LVFS were markedly reduced in the TAC group, while LVEDV, LVESV, LVIDd, and LVIDs were significantly increased, suggesting an impairment (such as organ ischemia, and hypoxia) of cardiac function. There was no apparent difference among Sham groups, or between the TAC and TAC + AGEs group. A striking improvement was seen in TAC + RAGE^−/−^ group compared with the TAC group (Fig. 1).

### RAGE knockout attenuates cardiac fibrosis through mediating EndMT in HF mice

**Fig. 2:**
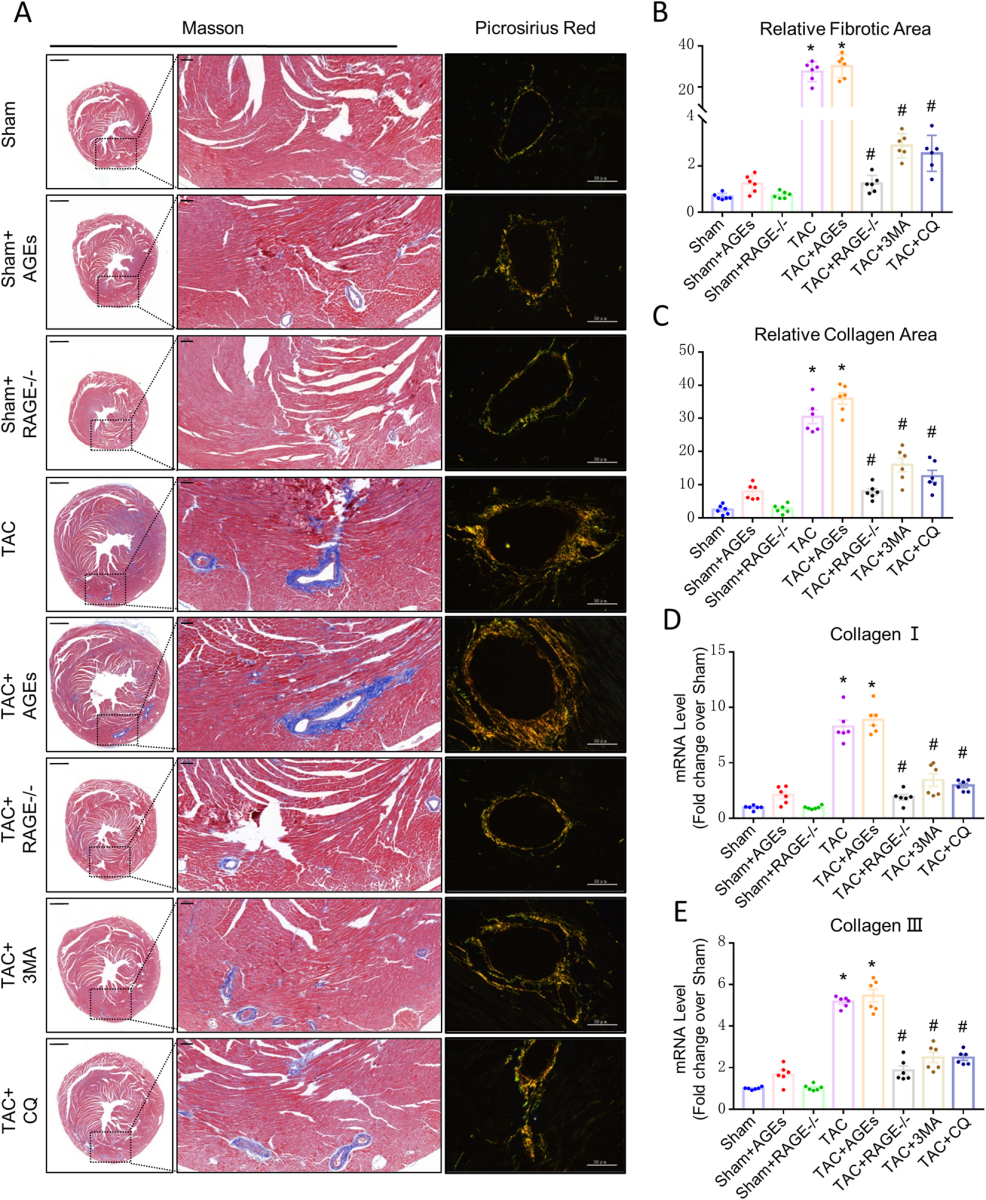
Effect of AGEs/RAGE or autophagy on cardiac fibrosis after TAC. **A** Myocardial fibrosis was detected by Masson’s trichrome staining (scale bar: transverse sections = 1000 µm, perivascular sections = 200 µm), and perivascular collagen synthesis was detected by a Sirius red-polarized method (scale bar = 50 µm). Blue areas indicate fibrosis. Yellow areas indicate collagen I, and green areas indicate collagen III. The fibrosis area was measured with a quantitative digital image analysis system: fibrotic area (**B**) and collagen deposition area (**C**). mRNAs for fibrosis associated genes collagen I (**D**) and collagen III (**E**) were measured by qPCR. *n* = 6. Data are presented as mean ± SEM. *p < 0.05 vs. sham group, ^#^*p* < 0.05 vs. TAC group.

On histological examination, the TAC group showed marked fibrosis in the myocardium, especially in the perivascular area, relative to Sham controls. RAGE knockout prevented increased fibrogenesis both in the myocardium and perivascular area (Fig. 2A, B). On exploring perivascular collagen deposition, we found that COL 1 and collagen type III (COL 3) accumulated in TAC group, and COL 1 increased significantly over COL 3. In contrast, there was obvious reduction in both COL 1 and COL 3 in RAGE knockout mice (Fig. 2A, C–E). There was no significant difference among Sham groups or the TAC and TAC + AGEs group.

**Fig. 3:**
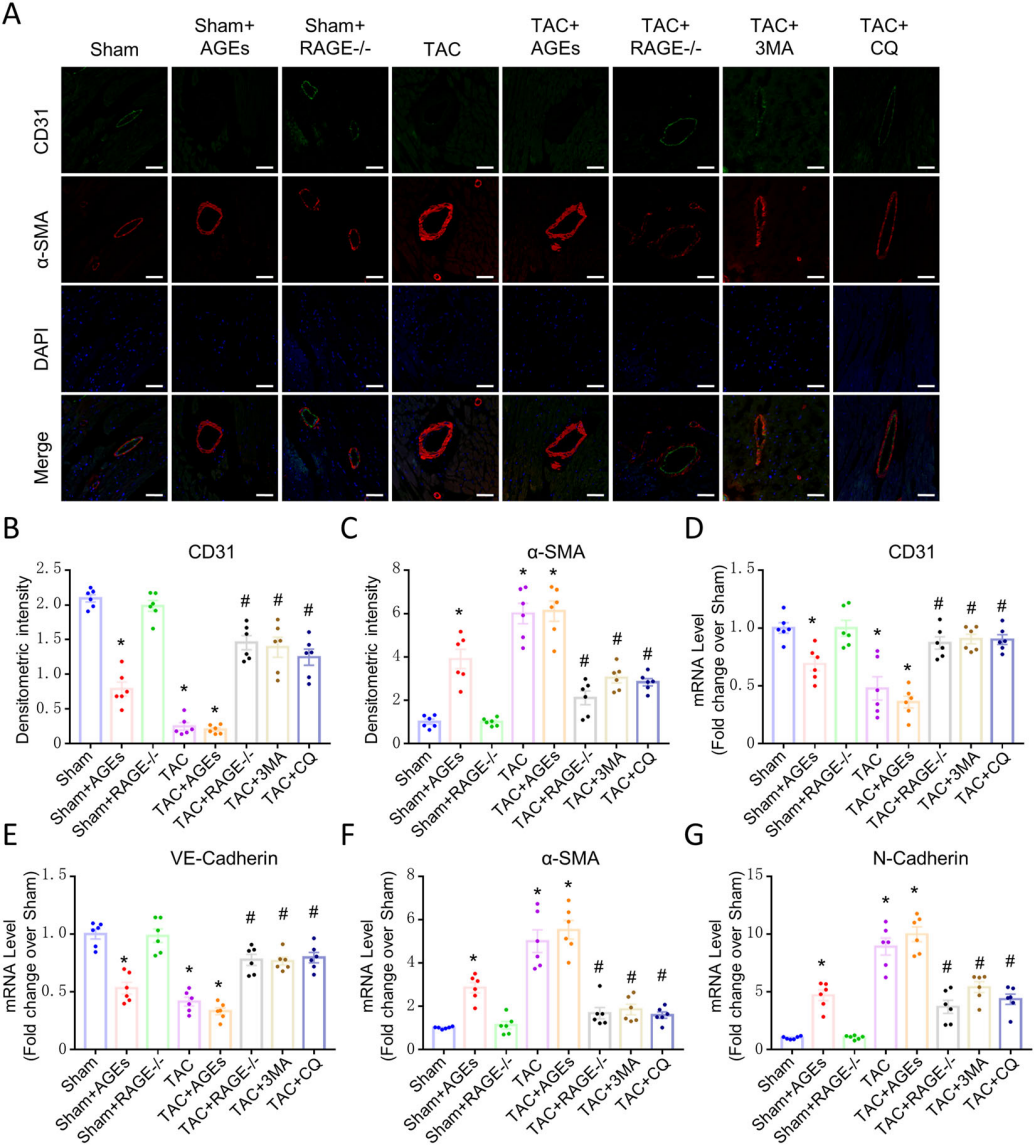
Endothelial cells may transition to mesenchymal cells in HF. **A** Confocal microscopic image of double immunofluorescence staining with CD31 (green) and α-SMA (red); nuclei were counter stained with DAPI (blue); Scale bars = 100 µm. **B**, **C** Quantification of immunofluorescence analysis. mRNAs for endothelial cells-associated genes [CD31 (**D**), VE-Cadherin (**E**)] and mesenchymal cells-associated gene [α-SMA (**F**) and N-Cadherin (**G**)] were measured by qPCR. *n* = 6. Data are presented as mean ± SEM. *p < 0.05 vs. sham group, ^#^*p* < 0.05 vs. TAC group.

As the perivascular area was most severely affected by fibrosis, we hypothesized that EndMT contributes to cardiac fibrosis in HF mice. We performed double immunofluorescence staining with antibodies to CD31 and α-SMA and found that CD31 was markedly down-regulated in myocardial vessels in the TAC group and mice treated with AGEs, while this down-regulation was reversed upon RAGE knockout. In contrast, α-SMA was up-regulated in the myocardial vessels of the TAC group, TAC + AGEs group and Sham + AGEs group; however, this up-regulation was also reversed in RAGE knockout mice (Fig. 3A–C). Expression of CD31 and VE-Cadherin (markers of endothelial cells) were decreased, whereas expression of α-SMA and N-Cadherin (markers of mesenchymal cells) were increased in HF mice and mice treated with AGEs (Fig. 3D–G). Again, RAGE knockout reversed these mRNA changes.

**Fig. 4:**
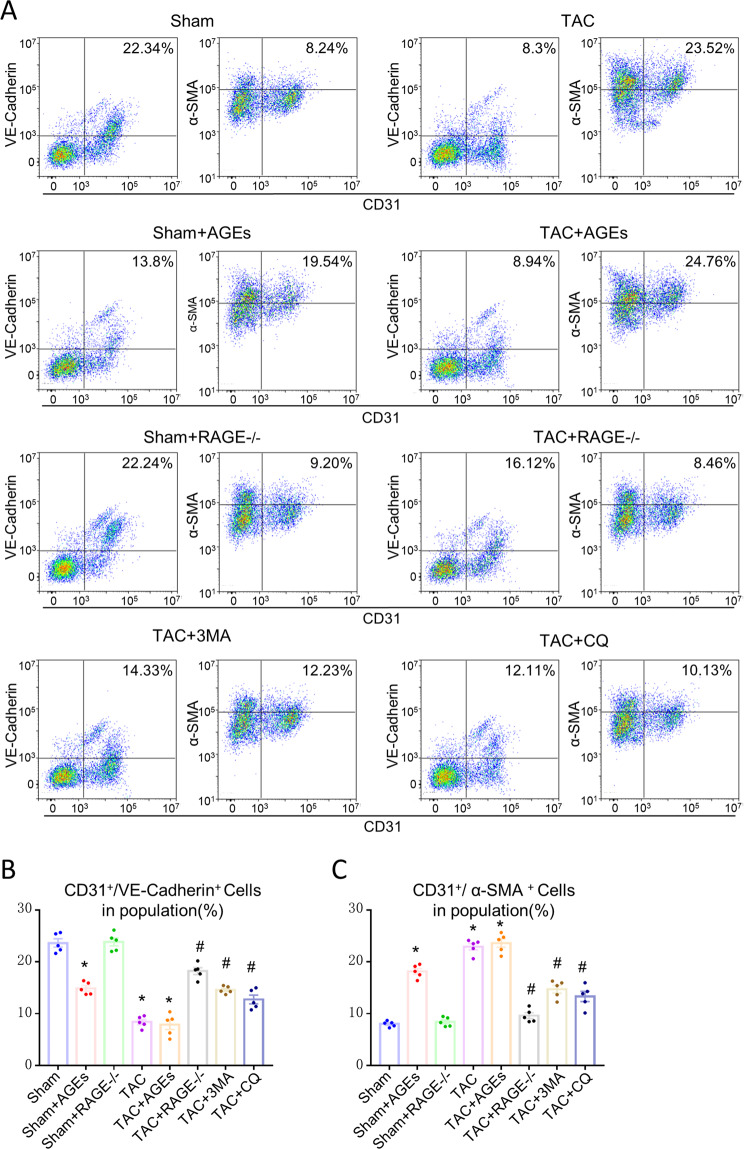
EndMT is one origin of myofibroblasts in HF and could be regulated by AGEs/RAGE and autophagy. **A** Flow cytometry analysis of primary isolates of mouse left ventricular cells. After mechanical disaggregation and enzymatic digestion, single-cell suspensions were incubated with combination of fluorescent antibodies to CD31, VE-Cadherin, and α-SMA. **B**, **C** Quantification of co-expressed cell analysis. *n* = 5. Data are presented as mean ± SEM. *p < 0.05 vs. sham group, ^#^*p* < 0.05 vs. TAC group.

Flow cytometry was used to study whether EndMT is one origin of myofibroblasts in HF. Compared with the Sham control, there was a 2.69-fold decrease in the number of CD31^+^/VE-Cadherin^+^ endothelial cells, and about a 2.85-fold increase in the number of CD31^+^/α-SMA^+^ (α-smooth muscle actin, myofibroblast marker) cells in the TAC group. Only 8.24% cells co-expressed CD31 and α-SMA, while about 22.34% cells co-expressed CD31 and VE-Cadherin in the Sham group. Moreover, AGEs/RAGE regulated the EndMT, as shown in Fig. 4. Compared with the Sham control, there was a 1.62-fold decrease in the number of CD31^+^/VE-Cadherin^+^ endothelial cells and about a 2.37-fold increase in the number of CD31^+^/α-SMA^+^ cells in the Sham + AGEs group, while compared with the TAC group, there was a 1.94-fold increase in the number of CD31^+^/VE-Cadherin^+^ endothelial cells, and about a 2.78-fold decrease in the number of CD31^+^/α-SMA^+^ cells in the TAC + RAGE^−/−^ group, but no difference in the TAC + AGEs group.

### AGEs/RAGE could regulate autophagy

**Fig. 5:**
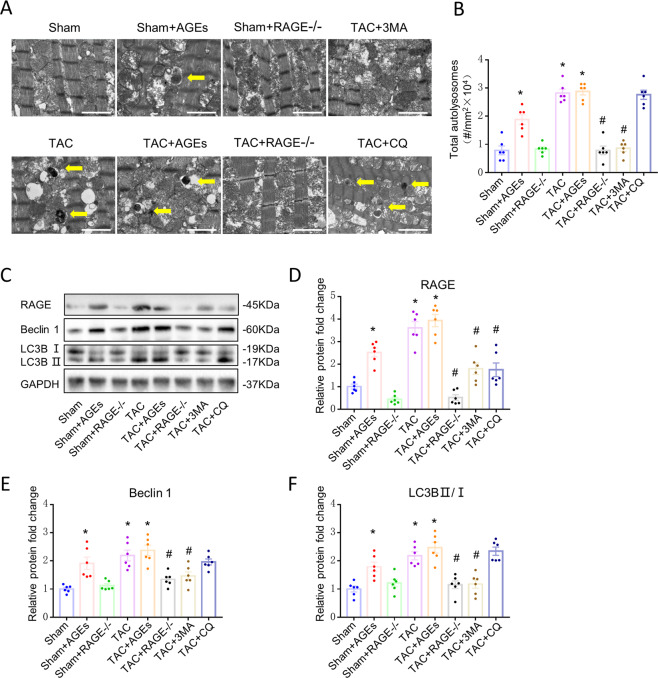
Reduction of autophagy at 8 weeks after TAC by genetic deletion of RAGE or an autophagy inhibitor (3-MA or CQ). **A** Representative autophagic ultrastructure of the heart tissue under transmission electron microscopy (Scale bars = 2 µm; arrow: autolysosome). **B** Quantification of autophagy lysosomes analysis. **C**–**F** Left ventricular levels of RAGE and autophagy-related proteins (LC3BII/I and Beclin 1) were assessed by western blot. Quantification of RAGE (**D**), Beclin 1 (**E**), and LC3BII/I (**F**). GAPDH was used as the internal control. *n* = 6. Data are presented as mean ± SEM. *p < 0.05 vs. sham group, ^#^*p* < 0.05 vs. TAC group.

Autolysosomes of cardiac tissues were analyzed by TEM. Increased typical autolysosomes were detected in the TAC group, TAC + AGEs group, and Sham + AGEs group, while Sham controls showed normal myocardial fine structure. Like the autophagy inhibitors, RAGE knockout inhibited autophagic activity (Fig. 5A, B). Western blot showed increased expression of proteins related to autophagy (LC3BII/I and Beclin 1) in the TAC group, TAC + AGEs group, and Sham + AGEs group. However, LC3BII/I and Beclin 1 protein expression levels were significantly lower in the TAC + RAGE^−/−^ group compared with the TAC group (Fig. 5C–F).

### Autophagy inhibitors alleviate cardiac fibrosis through mediating EndMT

To examine whether autophagy could regulate EndMT, we treated mice with 3-MA (TAC + 3-MA) and CQ (TAC + CQ). Echocardiography results (Fig. 1) and histological examination (Fig. 2) suggested that inhibition of excessive autophagy ameliorated cardiac fibrosis and cardiac function in HF. 3-MA and CQ mice both prevented the down-expression of CD31 and VE-Cadherin, the up-expression of α-SMA and N-Cadherin at mRNA level (Fig. 3), as well as the decrease in CD31^+^/VE-Cadherin^+^ endothelial cells and increase in CD31^+^/α-SMA^+^ cells (Fig. 4), implying that autophagy may mediate EndMT in HF.

### AGEs/RAGE mediated EndMT by regulating autophagy activity

**Fig. 6:**
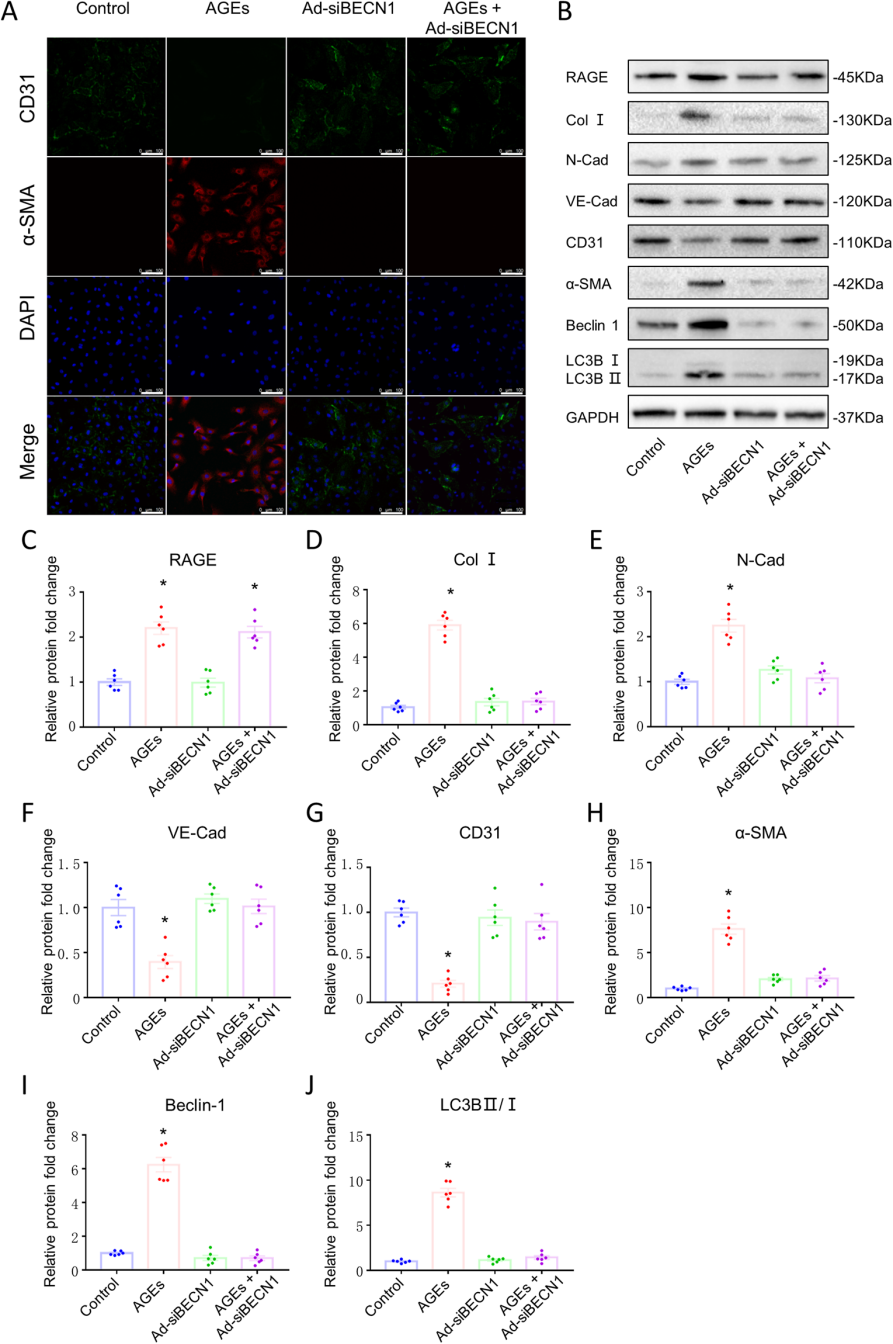
AGEs-RAGE induced EndMT by mediating endothelial autophagy in HUVECs. **A** Confocal microscopic image of double immunofluorescence staining for CD31 (green) and α-SMA (red); nuclei were counter stained with DAPI (blue); (Scale bars = 100 µm). **B** Protein expression of Beclin 1, LC3BII/I, RAGE, Collagen I, N-Cadherin, VE-Cadherin, CD31, and α-SMA assessed by western blotting. Quantification of RAGE (**C**), COL I (**D**), N-Cadherin (**E**), VE-Cadherin (**F**), CD31 (**G**), α-SMA (**H**), Beclin 1 (**I**), and LC3BII/I (**J**). GAPDH was used as the internal control. *n* = 6. Data are presented as mean ± SEM. *p < 0.05 vs. control group.

To clarify the regulatory mechanisms of EndMT, we cultured HUVECs and performed Ad-siRNA transfection to knockout BECN1 expression in the cultured cells. Treatment with 200 µg/ml AGEs for 24 h was optimal to induce EndMT of HUVECs, reflected in loss of cell–cell junctions, cobblestone morphology, and translation into long filopodia characteristic of mesenchymal cells. The expression of mesenchymal cells were significantly up-regulated and endothelial cells were down-regulated, according to the mRNA level (Supplementary Fig. 1) and the protein expression level (Fig. 6B, D–H). Interestingly, Beclin 1 knockout prevented EndMT in HUVECs subjected to AGEs (Fig. 6). Then, we detected the cell contractility using a three-dimensional collagen lattice and found that AGEs treatment significantly increased cell contractility after 24 h, with gel size reduced to 50% compared to controls. Beclin 1 knockout and RAGE knockout significantly repressed the cell contraction induced by AGEs (Supplementary Fig. 2). These findings suggest that AGEs/RAGE may mediate EndMT by regulating autophagy activity.

## Discussion

The present study investigated the regulatory mechanisms of EndMT in cardiac fibrosis of overload-induced HF. We found that EndMT may be the source of myofibroblasts and showed that AGEs could induce EndMT and autophagy. In addition, our data showed RAGE knockout could effectively inhibit autophagy, reduce EndMT, ameliorate myocardial fibrosis and cardiac function in HF mice. Autophagy may also be a crucial mediator between AGEs/RAGE and EndMT. Autophagy inhibitors attenuated EndMT as well as cardiac fibrosis and cardiac function. Beclin 1 knockout could directly eradicate AGEs/RAGE-induced EndMT in HUVECs, thus providing useful insights into the AGEs/RAGE-autophagy-EndMT axis as a promising target for the treatment of HF.

Recently, AGEs/RAGE has been regarded as an effective clinical prognostic index of cardiovascular disease, with AGEs level positively correlated with postinfarction HF development^39^. High sRAGE has been associated with deteriorating left ventricular function and an increased rate of HF post-discharge hospitalization^40–42^. Our study showed that RAGE knockout could prevent cardiac fibrosis and COL 1 deposition in TAC-induced HF mice. It has been reported that endomyocardial fibrosis is associated with selective deposition of COL 1^43,44^, which is consistent with our results.

Importantly, we found that RAGE knockout could down-regulate expression of α-SMA and N-Cadherin, and up-regulate expression of CD31 and VE-Cadherin. VE-Cadherin is a component of endothelial cell-to-cell adherens junction^45^, whereas N-Cadherin increases cell separation and cell motility. The switch from VE-Cadherin to N-Cadherin suggested the occurrence of EndMT in TAC-induced HF mice^46^. We also observed increased co-expression of CD31 and α-SMA in cardiac cells. α-SMA is characteristic of activated myofibroblasts involved in pathological cardiac remodeling and subsequent cardiac fibrosis in HF^3^. Accordingly, our results suggest that RAGE knockout could inhibit EndMT and EndMT may be one of the vital sources of myofibroblasts involved in cardiac fibrosis in HF. Previous studies have identified AGEs as triggers of EndMT^35,47–49^. Our results suggested that AGEs could induce EndMT in vivo and in vitro. However, EndMT induced by AGEs did not cause cardiac fibrosis and influence cardiac function in Sham mice treated with AGEs, which may be a compensatory effect under physiological conditions, while RAGE knockout effectively improve cardiac function, reduce EndMT and prevent myocardial fibrosis in HF mice. Therefore, AGEs/RAGE mediated EndMT may be a potential target for treating cardiac fibrosis in HF.

Consistent with our previous study^30^, we identified AGEs/RAGE as a positive regulator of autophagy. Although basal autophagy is important in cells^50^, excessive autophagy is harmful and has a deleterious effect in various diseases^51–53^. Autophagy has become a cardiovascular therapeutic target^54,55^, with growing evidence that inhibition early autophagosome formation could decrease autophagy, resulting in diminishing pathological remodeling induced by severe pressure stress^56,57^. Our study confirmed that 3-MA (the inhibitor of autophagosome formation) and CQ (the blocker of autolysosome degradation) both protected against cardiac fibrosis and reduced EndMT in HF mice at 8 weeks. These findings suggest that excessive autophagic flux may result in cardiac fibrosis via EndMT.

Importantly, our study identified that AGEs/RAGE mediated EndMT by regulating autophagy activity. Autophagic cell death has been shown in HUVECs treated after 24 h with glycated collagen, a significant component of AGEs^58^. Our study detected obvious expression of mesenchymal cells, collagen deposition, decreased expression of endothelial markers, and enhanced cellular contractility after AGEs treatment for 24 h. However, the transition was inhibited when autophagy was blocked, suggesting that AGEs/RAGE contribute to EndMT-induced cardiac fibrosis through regulating autophagy in HF.

In summary, autophagy mediated by AGEs/RAGE triggers EndMT in the progression of HF. RAGE knockout could inhibit autophagy, thus resulting in prevention of EndMT, as well as attenuating cardiac fibrosis and improving cardiac function. AGEs/RAGE-autophagy-EndMT axis could be an attractive target for HF treatment.

## Supplementary information

Supplementary material

supplementary figure 1

supplementary figure 2
