# Supplementary material for: Knockout RAGE alleviates cardiac fibrosis through repressing endothelial-to-mesenchymal transition (EndMT) mediated by autophagy
Source: Cell Death Dis. 2021 May 11;12(5):470. doi: 10.1038/s41419-021-03750-4 (PMC8113558; doi:10.1038/s41419-021-03750-4)
Supplement: Supplementary file 1 — Supplementary material [file 41419_2021_3750_MOESM1_ESM.docx]

**SUPPLEMENTARY FIGURE 1 | EndMT occurred under AGEs treatment (200 µg/ml) for 24 h in HUVECs.** (A) Changes in endothelial morphological phenotype induced by AGEs (Scalebars = 100 µm). (B–E) mRNAs for endothelial cells-associated gene [CD31, VE-Cadherin] and mesenchymal cells-associated gene [α-SMA and N-Cadherin] were measured under different durations (B, D) and concentrations (C, E) of AGEs by qPCR. GAPDH was used as the internal control. n = 6. Data are presented as mean ± SEM. ^∗^p< 0.05 vs. Control group.

**SUPPLEMENTARY FIGURE 2 | Inhibition of RAGE and autophagy could repress cellular contractility induced by AGEs in HUVECs.** (A)The presentative photomicrographs (0 h and 24 h) of collagen gel contraction assay. (B) Summary of contractility results in terms of diameter of the gel surface. n = 6. Data are presented as mean ± SEM.^∗^p < 0.05 vs. Control group.
