# Supplementary figures and images for: Knockout RAGE alleviates cardiac fibrosis through repressing endothelial-to-mesenchymal transition (EndMT) mediated by autophagy
Source: Cell Death Dis. 2021 May 11;12(5):470. doi: 10.1038/s41419-021-03750-4 (PMC8113558; doi:10.1038/s41419-021-03750-4)

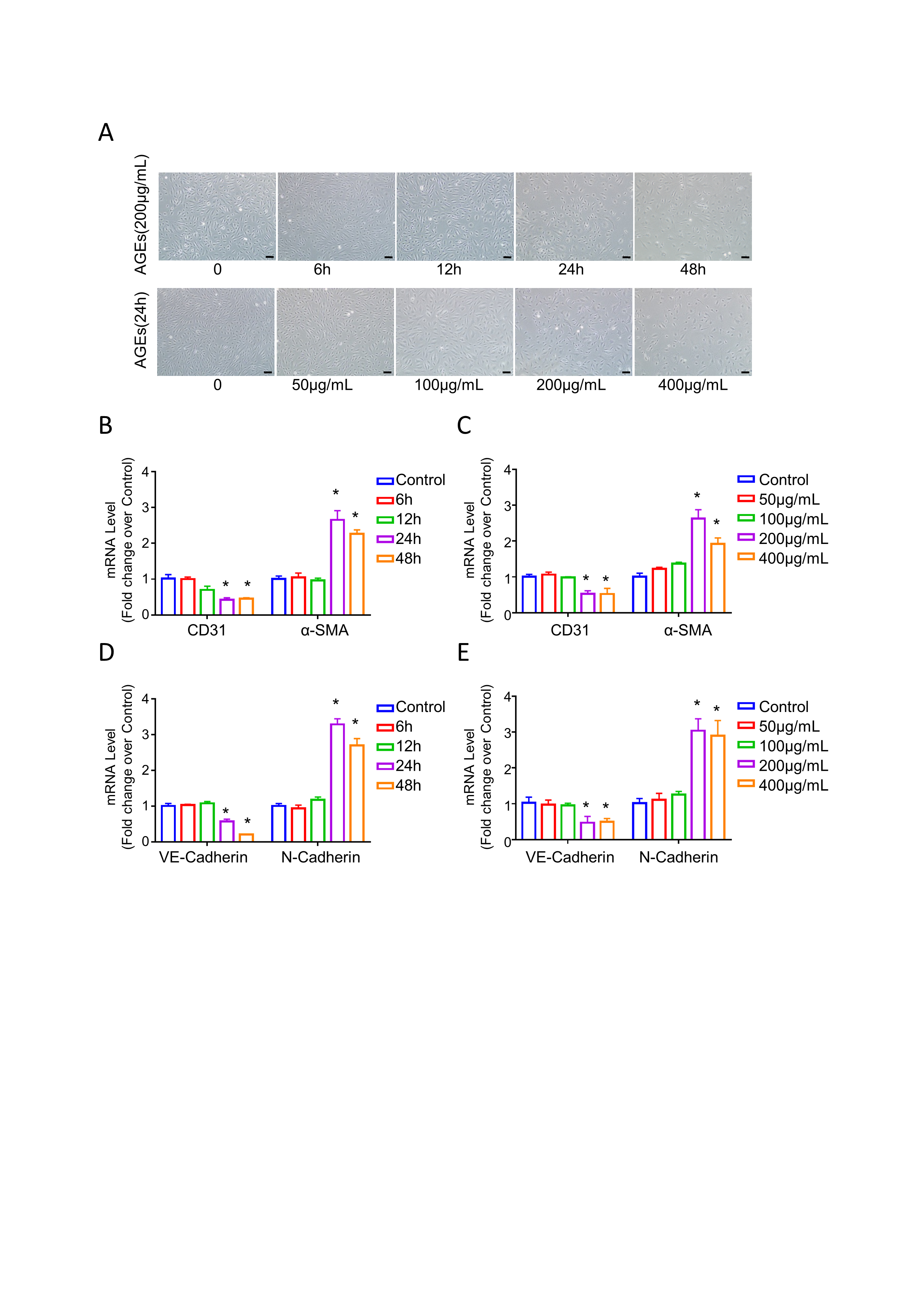

Supplement: Supplementary file 2 — supplementary figure 1 [file 41419_2021_3750_MOESM2_ESM.tif]

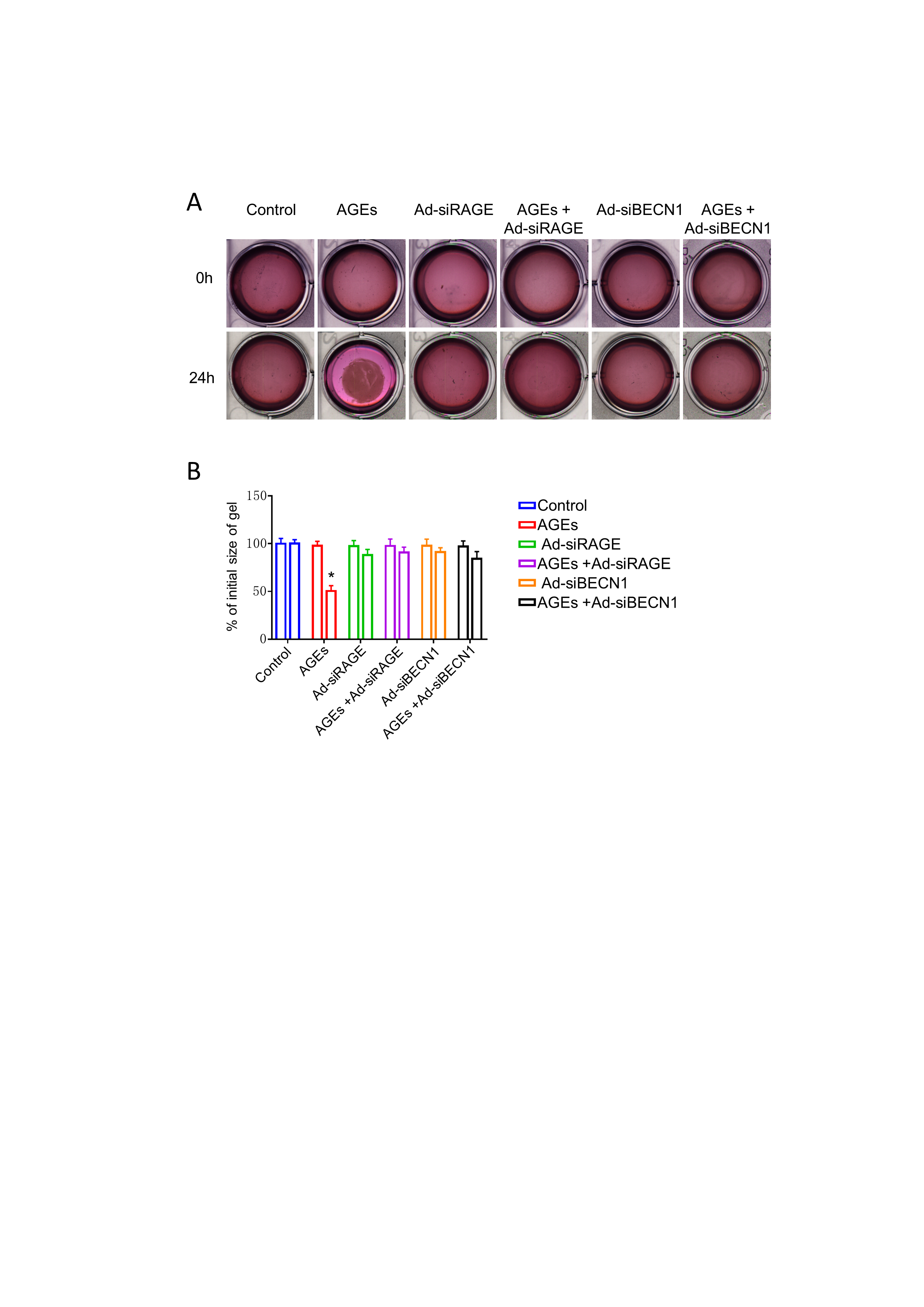

Supplement: Supplementary file 3 — supplementary figure 2 [file 41419_2021_3750_MOESM3_ESM.tif]
